# Complete Genome Sequence of *Bacillus megaterium* Podophage Palmer

**DOI:** 10.1128/genomeA.00358-15

**Published:** 2015-05-07

**Authors:** Emily C. Hargrove, Mariana S. Lopez, Adriana C. Hernandez, Gabriel F. Kuty Everett

**Affiliations:** Center for Phage Technology, Texas A&M University, College Station, Texas, USA

## Abstract

*Bacillus megaterium* has been widely used as a research tool for decades. Its use is on the rise as a recombinant protein production host and as a bioremediation bacterium. Bacteriophages against this bacterium may have biotechnological applications. Here, we describe the novel podophage Palmer, which infects *B. megaterium*.

## GENOME ANNOUNCEMENT

*Bacillus megaterium* is a Gram-positive bacterium widely used in industry as a protein production host (1). Information can be gleaned from the study of bacteriophages which infect this host to improve the use of the bacterium in both research and industrial settings. For that purpose, novel podophage Palmer was isolated and characterized.

Bacteriophage Palmer was isolated from a soil sample collected in College Station, TX, USA. Phage DNA was sequenced in an Illumina MiSeq 250-bp paired-end run with a 550-bp insert library at the Genomic Sequencing and Analysis Facility at the University of Texas (Austin, TX, USA). Quality controlled, trimmed reads were assembled to a single contig of circular assembly at 117.8-fold coverage using Velvet version 1.2.10. The contig was confirmed to be complete by PCR using primers that face the upstream and downstream ends of the contig. Products from the PCR amplification of the junctions of concatemeric molecules were sequenced by Sanger sequencing (Eton Bioscience, San Diego, CA). Genes were predicted using GeneMarkS (2) and corrected using software tools available on the Center for Phage Technology (CPT) Galaxy instance (https://cpt.tamu.edu/galaxy-public/). The morphology of Palmer was determined using transmission electron microscopy performed at the Texas A&M University Microscopy and Imaging Center.

Palmer has a 40,000-bp genome with 50 predicted coding sequences, a G+C content of 40.67%, and a coding density of 95.9%. Palmer shares a 66.7, 86.7, 87.6, and 87.8% identity with *B. megaterium* podophages Pascal (GenBank accession no. KM236247), Page (accession no. NC_022764), Pookie (accession no. KM236248), and Pony (accession no. NC_022770), respectively, as determined by Emboss stretcher (3).

Several phage replication and recombination genes were identified, including two single-strand DNA-binding proteins (one RecT-like), a DnaD/DnaB-like primosome protein, a DnaC-like replication protein, and a plasmid replication/relaxation protein. Homologs of the plasmid replication/relaxation protein are found in several *Bacillus* phages although their role in the phage life cycle is currently unknown. Also present are an RNA polymerase sigma factor and three transcriptional regulators. One transcriptional regulator is a lambda repressor-like protein indicating that Palmer may be a temperate phage. Genes encoding structural proteins were identified, including a capsid protein, tail spike, and a tail fiber. The tail spike protein contains a pectin lyase domain, presumably to degrade biofilm and promote infection (4). Other genes encoding structural proteins were identified by sequence homology. To accomplish DNA packaging, Palmer contains a head-to-tail joining protein and small and large terminase proteins. TerL homology to other phages indicates that Palmer uses a *pac*-type head-full packaging. By precedent, the genome was opened to the small terminase gene for annotation purposes (5). To accomplish host cells lysis, Palmer encodes a phi29-like holin, a putative antiholin, and two peptidases. Palmer also has an FtsK/SpoIIIE protein, which is usually associated with DNA translocation in sporulating cells, including *B. megaterium* (6, 7). Palmer may use this protein to regulate the injection of its DNA into the host cell.

### Nucleotide sequence accession number. 

The genome sequence of phage Palmer was contributed as accession no. KP411017 to GenBank.
